# MRI network progression in mesial temporal lobe epilepsy related to healthy brain architecture

**DOI:** 10.1162/netn_a_00184

**Published:** 2021-04-27

**Authors:** Victoria L. Morgan, Graham W. Johnson, Leon Y. Cai, Bennett A. Landman, Kurt G. Schilling, Dario J. Englot, Baxter P. Rogers, Catie Chang

**Affiliations:** Institute of Imaging Science, Department of Radiology and Radiological Sciences, Vanderbilt University Medical Center, Nashville, TN, USA; Department of Biomedical Engineering, Vanderbilt University, Nashville, TN, USA; Department of Biomedical Engineering, Vanderbilt University, Nashville, TN, USA; Department of Electrical Engineering and Computer Science, Vanderbilt University, Nashville, TN, USA; Institute of Imaging Science, Department of Radiology and Radiological Sciences, Vanderbilt University Medical Center, Nashville, TN, USA; Department of Neurological Surgery, Vanderbilt University Medical Center, Nashville, TN, USA; Institute of Imaging Science, Department of Radiology and Radiological Sciences, Vanderbilt University Medical Center, Nashville, TN, USA; Department of Electrical Engineering and Computer Science, Vanderbilt University, Nashville, TN, USA

**Keywords:** Network connectivity, Focal epilepsy, Functional MRI, Diffusion MRI

## Abstract

We measured MRI network progression in mesial temporal lobe epilepsy (mTLE) patients as a function of healthy brain architecture. Resting-state functional MRI and diffusion-weighted MRI were acquired in 40 unilateral mTLE patients and 70 healthy controls. Data were used to construct region-to-region functional connectivity, structural connectivity, and streamline length connectomes per subject. Three models of distance from the presumed seizure focus in the anterior hippocampus in the healthy brain were computed using the average connectome across controls. A fourth model was defined using regions of transmodal (higher cognitive function) to unimodal (perceptual) networks across a published functional gradient in the healthy brain. These models were used to test whether network progression in patients increased when distance from the anterior hippocampus or along a functional gradient in the healthy brain decreases. Results showed that alterations of structural and functional networks in mTLE occur in greater magnitude in regions of the brain closer to the seizure focus based on healthy brain topology, and decrease as distance from the focus increases over duration of disease. Overall, this work provides evidence that changes across the brain in focal epilepsy occur along healthy brain architecture.

## Author Summary

In patients with focal epilepsy, seizures originate in the focus and propagate across the brain. Over years of duration of disease, these repeated seizures lead to network reorganization and disruption. We hypothesized that these changes occur along a framework that could be identified through healthy brain architecture, with the greatest changes occurring closest to the seizure focus and decreasing as distance from the focus increases. In this work we detected this pattern of change in functional and structural networks when distance to the focus was measured by functional and structural connectivity, respectively. Overall, this work presents a framework of spatiotemporal network progression over duration of disease related to the seizure focus and healthy brain architecture that may predict individual network evolution in focal epilepsy.

## INTRODUCTION

Approximately 60%–70% of focal epilepsy patients are drug-refractory (Laxer et al., 2014; Engel, 2016). Many of these patients experience seizures for decades, prior to surgical or other intervention (Engel, 2016). Evidence suggests that epilepsy is not a static condition, and that this progression can have significant clinical implications. A recent review concluded that patients with shorter epilepsy duration are more likely to be seizure free after surgical resection (Bjellvi et al., 2019). Similarly, shorter duration of disease predicts better seizure outcome according to several prediction algorithms (Gracia et al., 2015; Jehi et al., 2015; Dugan et al., 2017). This implies that our current surgical treatment strategies do not successfully consider the issue of temporal progression in epilepsy. Focal epilepsy also has spatially extensive effects on the brain evidenced by widespread functional (Englot et al., 2016) and structural (Bonilha et al., 2012; Besson et al., 2014) network connectivity changes measured by MRI. Network connectivity has been shown to be related to seizure outcome after surgery (Morgan et al., 2017; Gleichgerrcht et al., 2018; Taylor et al., 2018; Morgan et al., 2019) and correlated with duration of disease (Morgan et al., 2011; Haneef et al., 2015; Wang et al., 2017; Owen et al., 2020). Thus, the progression of focal epilepsy includes a complex spatiotemporal interaction of widespread brain networks, and this evolution of network architecture may play a significant role in the efficacy of treatment.

To be able to utilize the spatiotemporal network information as a biomarker of treatment outcome prediction, it is important to model these network changes in the context of a large-scale overarching organization of the brain. This would (a) allow the ability to predict changes in an individual patient to facilitate more accurate outcome predictions and (b) improve the understanding of how focal epilepsy progresses over time and space across the brain. Various topological structures have been proposed to explain variability in brain network connectivity in healthy controls (Suárez et al., 2020). For example, some relate functional relationships across the brain in terms of structural connectivity (van den Heuvel et al., 2009), communication measures (Goñi et al., 2014; Fukushima et al., 2018), microstructural characteristics (Vázquez-Rodríguez et al., 2020), or geometric properties (Betzel et al., 2016; Oligschläger et al., 2017). In temporal lobe epilepsy, Larivière et al. investigated the functional connectivity differences in patients as they relate to geodesic distance between cortical vertices (Larivière et al., 2020). Overall, they found increases in short-range and decreases in long-range functional connectivity in the temporal and frontal lobes mediated by white matter microstructural changes measured by diffusion MRI. Furthermore, reductions in short-range connectivity were related to better surgical outcomes. These findings support that pathological changes in focal epilepsy are not random, but rather progress along an identifiable framework and may have clinical significance.

In this work we investigated brain organization models specifically related to unilateral mesial temporal lobe epilepsy (mTLE) as a framework on which MRI functional and structural connectivity changes occur over time and space in these patients. In mTLE, seizures originate within one hippocampus and propagate across the brain (Engel, 2001). Therefore, our proposed models were based on the general assumption that greater network changes will occur “closer” to the hippocampus based on some topology, and then spread outward. We presented two separate investigations. First, we limited our network to regional connections to the hippocampi (presumed seizure focus). Second, we investigated whole-brain connectomes with each region connected to all other regions in the brain. If functional and structural network evolution in mTLE can be modeled using a framework defined in the healthy brain, a similar approach may be used to predict progression in other focal epilepsies.

## MATERIALS AND METHODS

### Participants

Participants were 40 drug-refractory unilateral mTLE patients (29 right, 11 left) prior to surgical intervention (Table 1). Diagnoses were based on long-term video EEG of ictal events localizing to anterior/mesial temporal regions, unilateral mesial temporal lobe hypometabolism on positron emission tomography (PET), hippocampal sclerosis on standard MRI, and seizure semiology consistent with mTLE. Two patients did not have hippocampal sclerosis on clinical MRI or pathology-confirmed gliosis in the resected tissue. One of these had resection with an Engel I-d outcome 1 year postsurgery, while one did not have resection. Exclusion criteria included structural abnormalities outside the mesial temporal lobe. In addition, 70 healthy control participants were enrolled. The protocol was approved by the Vanderbilt University Institutional Review Board. All participants gave informed consent.

**Table 1.** Participant characteristics.

	**Right mTLE (*n* = 29)**	**Left mTLE (*n* = 11)**	**Controls (*n* = 70)**
M/F	14/15	8/3	37/33
Age (years: mean ± *SD*, min, max)	40.1 ± 10.7, 23, 62	36.6 ± 16.4, 18, 68	38.0 ± 13.7, 18, 71
Epilepsy duration (years: mean ± *SD*, min, max)	21.2 ± 14.4, 2, 50	20.6 ± 16.2, 3, 46	
MRI HS or hippocampal gliosis on pathology (*n*, %)	27, 93%	13, 100%	
Lateralizing PET hypometabolism (*n*, %)	24, 83%	8, 73%	
Localizing video scalp EEG of ictal events (*n*, %)	25, 86%	10, 91%	

*Note*. M = male; F = female; HS = hippocampal sclerosis including T2 hyperintensity.

### Imaging

Imaging was identical for all participants and was acquired on a 3T MRI scanner using a 32-channel head coil. Cardiac and respiratory fluctuations were recorded at 500 Hz using the scanner-integrated pulse oximeter and respiratory belt. The following images were acquired: (a) high-resolution T1-weighted MRI for intersubject normalization and regional and tissue segmentation (1 mm × 1 mm × 1 mm), (b) T1-weighted MRI acquired in the same slice orientation as the functional images (1 mm × 1 mm × 3.5 mm with 0.5-mm gap), (c) T2*-weighted functional MRI (fMRI) BOLD MRI at rest with eyes closed (34 axial slices, echo time = 35 ms, repetition time = 2 sec, 3 mm × 3 mm × 3.5 mm with a 0.5-mm gap, 10 minutes), and (d) diffusion-weighted MRI (DWI) for structural connectivity (50 slices, 2.5 mm × 2.5 mm × 2.5 mm, 92 directions, b = 0, 1,600 s/mm^2^).

### Connectomes

A total of 109 regions of interest were identified on the 1-mm^3^ T1-weighted images, including 54 in each hemisphere and the bilateral brainstem. First, the Multi-Atlas algorithm (Asman & Landman, 2013; Huo et al., 2016) was used to identify cortical and subcortical regions across the brain. Since this atlas did not parcellate subregions of the hippocampus, we then used the FreeSurfer 6 suite (Fischl, 2012) to identify hippocampal subfields. These were used to form composite anterior and posterior hippocampal regions according to McHugo et al. (Plassard et al., 2017; McHugo et al., 2018). Overall, this parcellation is relatively coarse to reduce dimensions in our analyses and to account for lower spatial sampling of functional MRI data relative to anatomic imaging.

The fMRI images were preprocessed using SPM12 software (http://www.fil.ion.ucl.ac.uk/spm/software/spm12/) and MATLAB 2019a (MathWorks, Natick, MA). First, physiological noise correction using the retrospective correction of physiological motion effects in functional MRI (RETROICOR) protocol (Glover et al., 2000) was implemented using the pulse oximeter and respiratory belt data. Next, SPM12 was used for slice timing correction, motion correction, spatial normalization to the Montreal Neurological Institute template via the T1-weighted datasets, and spatial smoothing (6 mm × 6 mm × 6 mm FWHM Gaussian kernel). Then the fMRI time series were temporally band-pass filtered at 0.0067 to 0.1 Hz (Cordes et al., 2001).

The preprocessed functional MRI time series were averaged across all voxels in each region of interest. Then a partial Pearson correlation between each pair of averaged time series was computed using six motion and one mean white matter time series as confounds. The correlation coefficients were then normalized using the Fisher *Z* transform (Fisher, 1915). This resulted in a matrix of pairwise functional connectivity values, which in this work will be generally referred to as a functional connectivity (FC) connectome. To account for the effects of age in this connectome, a linear relationship was assumed. Using only the healthy controls, the linear fit and the root mean squared error of the fit were computed for each pair of regions (edge). This edge-wise fit was then used to correct the FC in each subject, resulting in a connectome of the FC residuals after linear age regression (FC_res_, *Z* values) and in standard deviation from age-matched control when further divided by root mean squared error of the linear fit for that edge (FC_corr_). This correction was done in native left and right hemispheres, but patients were then transformed into ipsilateral and contralateral with respect to seizure focus for some analyses, where specified.

The DWI images were preprocessed using MRtrix3 (Tournier et al., 2019) including denoising (Cordero-Grande et al., 2019), eddy current and motion correction (Andersson & Sotiropoulos, 2016), and bias correction of B1 field inhomogeneity (Tournier et al., 2019). Then the response function was estimated for spherical deconvolution for estimation of fiber orientation distribution (Tournier et al., 2007). Next, SPM12 and MATLAB 2019a were used to generate the gray matter–white matter interface using the high-resolution T1-weighted image and the mean B0 image. Using MRtrix3, 2 × 10^7^ anatomically constrained probabilistic streamlines were generated through the white matter from this interface (Smith et al., 2012). The streamlines were then reduced to 1 × 10^7^ using spherical convolution–informed filtering to match the fiber orientation density integrals (SIFT2) (Smith et al., 2015). The 109 regions of interest were then used to create a connectome matrix of the streamline count between each pair of regions scaled by the inverse of the two region volumes as a measure of structural connectivity (SC). In addition, similar to the FC connectome, the SC connectomes were corrected for age by using linear fits of the healthy control data. However, to convert these data to a Gaussian distribution, a log transform was used prior to the fitting. This resulted in SC connectomes of residuals and standard deviation from age-matched control, SC_res_, and SC_corr_ for each subject. These were also computed in left and right hemispheres, but were then converted to ipsilateral and contralateral to seizure focus in specified analyses. Similarly, using MRtrix3, a connectome matrix of mean streamline length between each pair of regions was also created (LEN).

### Models of Distance in the Healthy Brain

We developed four models, each based on a different topology (*T*), in the healthy brain to which MRI network progression in patients can be compared. Three topologies studied were based on distance to the presumed seizure focus and constructed using the connectomes defined above: streamline length (*T*_*LEN*_), structural connectivity (*T*_*SC*_), and functional connectivity (*T*_*FC*_). For each, the designated connectomes were averaged across all healthy controls to yield the topology matrix of the healthy brain.

Rather than distance between regions reflected in the first three topologies, the fourth topology was created based on membership into functional connectivity resting-state network (*T*_*RSN*_). Distance here refers to the hierarchy of transmodal functional networks (default mode network, other higher cognitive networks) to unimodal functional networks (perceptual, primary sensory/motor networks) defined by a principal gradient of cortical organization in the human functional connectome by Margulies et al. (2016). Using this idea, four resting-state networks (Buckner et al., 2008; Uddin et al., 2019), each consisting of five bilateral regions, were identified and ordered as follows: (a) default mode network, (b) attention network, (c) primary visual network, and (d) motor/sensorimotor network. This topology matrix was designed by repeating rows where each position represents one region of interest with the value (0–4) indicating which of the four networks in which it is included (1–4, respectively, or 0 if none).

Then, similar to the work by Betzel et al. (2016), we developed models of distance in the healthy brain with respect to a given seed region and topology. Specifically, let *R* = [*R*_(1)_ … *R*_(*N*)_] be a binary vector encoding the selected seed region, with *R*_(*n*)_ = $\left\{\begin{array}{c} 0,region\;n\;is\;not\;seed\;\#\\ 1,\;region\;n\;is\;seed \end{array}\right.$, and let *T* be a topology matrix corresponding to one of *T*_*LEN*_, *T*_*SC*_, *T*_*FC*_, or *T*_*RSN*_ as defined above, with elements $\left[\begin{array}{ccc} T(1,1) & \cdots & T(1,N)\\ \vdots & \ddots & \vdots \\ T(N,1) & \cdots & T(N,N) \end{array}\right]$. Then the modeled distance from region n to each other region, under topology, *T*, is given by the vector:

RxT=[T(n,1),T(n,2)…T(n,N)] (1)

For the FC, SC, and LEN topologies, *N* = 55, which represents the regions across one hemisphere and the brainstem. Cross-hemisphere connections in many subjects, especially for the SC, were nonexistent and so were not included. For resting-state networks (RSN) topology, *N* = 109 to represent all the regions of interest. We used these models to test whether network progression in patients increased when distance from the anterior hippocampus or along a functional gradient in the healthy brain decreased.

### Hippocampal Network Change in Patients

**Figure 1.**
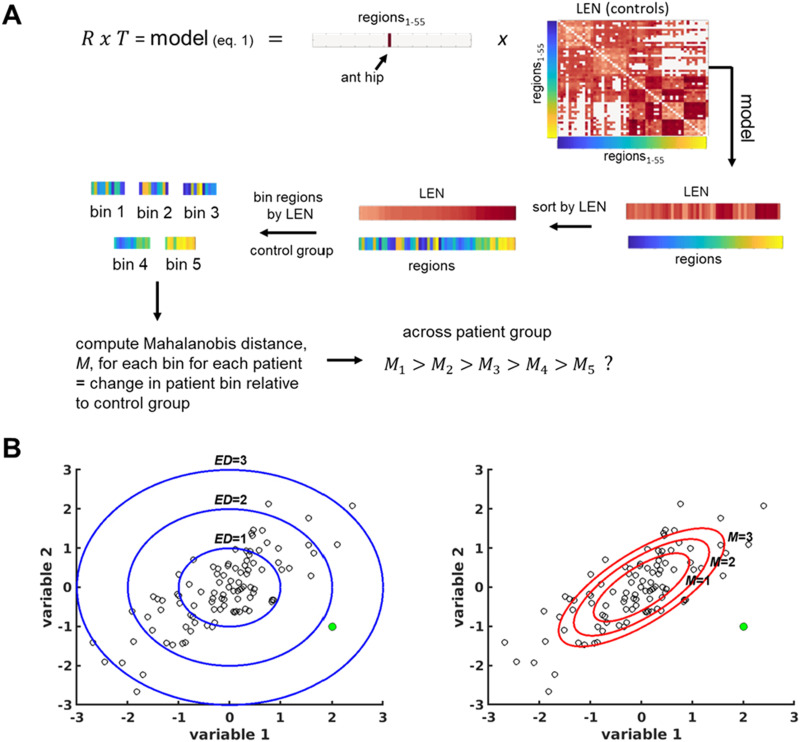
Hippocampal network change in patients. (A) Diagram representing analysis process using streamline length as the topology, *T*, and the anterior hippocampus (seizure focus) as the region of interest, *R*, to develop an example model of distance from *R* in the healthy brain. The model vector of LEN values is sorted and binned (in this example). The Mahalanobis distance is then computed for edges in each bin to quantify the patient’s change from the population of healthy controls. The hypothesis is that the Mahalanobis distance for the “closest” bin (bin 1, shortest LEN) will be highest, with decreasing values for bins of increasing LEN. (B) The Mahalanobis distance is a multivariate measure of change that accounts for the covariance between the variables, where the Euclidean distance does not. An example of 100 samples of two-variable data with a Pearson correlation of *r* = 0.74 and a mean at [0 0] is illustrated. The contours of Euclidean distance (*ED*) of 1, 2, and 3 are shown in blue (left), while the contours of the Mahalanobis distance (*M*) of 1, 2, and 3 are shown in red (right). An example point ([2, −1], shown in green) has an *ED* = 2.2 and *M* = 21.5; ant hip = anterior hippocampus; LEN = connectome based on streamline length across healthy controls.

Two types of MRI network progression were investigated and compared to the four models above. The first type of network was defined as the connection between the anterior hippocampus (presumed seizure focus) and each other region. To compare these hippocampal connections to a model, the following procedure was implemented (Figure 1A). The model vector from (Equation 1) was sorted into bins of regions based on distance defined by each topology. For the LEN model, the “closest” bin (bin 1) had the shortest length. For the FC model, bin 1 had the highest absolute value of the functional connectivity to the anterior hippocampus. For the SC model, bin 1 had the highest structural connectivity to the anterior hippocampus. For the RSN model, the edges were binned from their respective assignment to resting-state network based on transmodal to unimodal gradient (1–4). Thus, each bin consisted of approximately 10 edges between anterior hippocampus and other regions. Then, a multivariate *z*-score, Mahalanobis distance (De Maesschalck et al., 2000; Taylor et al., 2020) was used to compute network connectivity change between each patient and the control group across the edges in the given bin. The Mahalanobis distance, *M*, is a multivariate quantification of distance that, unlike Euclidean distance, considers the correlation between the variables (Figure 1A). As described in Taylor et al. (2020), we computed the Mahalanobis distance of the edges in a bin for a patient, M, using the following:

$$M=\sqrt{{\left(s-\mu \right)}^{T}\cdot C^{-1}\cdot (s-\mu )}$$ (2)

where *s* is the vector of length k of FC_res_ or SC_res_ values (after linear regression to account for age) of the edges in the bin in the patient, k is the number of edges in a bin, *μ*= the mean of the same FC_res_ or SC_res_ edges across a set of controls, and C is the covariance matrix between those measures in the set of controls computed using shrinkage estimators. To compare *M* across bins and patients, the number of edges (*k*) must be held constant across computations. Because some bins had 10 edges and some had 11, and because there were a few subjects in which some edges were not existent (mostly for SC), we performed 500 permutations with a random subset of 7 (out of 10 or 11) edges. Any sample in which the patient did not have seven detected edges was discarded. And, nested within this loop, we performed a second set of 500 permutations of a subset of 50 random control subjects (out of 70). Any loop with less than 45 controls having all seven edges was discarded. The mean of these permutations was used as the final *M*. These calculations are carried out in regions with respect to left and right in the brain in both patients and controls, and then converted to ipsilateral and contralateral with respect to the seizure focus to compare across patients. The analyses were performed with functional (*M*_*FC*_) and structural connectivity (*M*_*SC*_) across the edges separately to determine whether each type of connectivity is altered in patients as a function of distance to the anterior hippocampus or along a functional gradient in the healthy brain.

To test our hypothesis that *M* of bin 1 > bin 2 > bin 3 > bin 4 > bin 5, we used three statistics. First, there must be difference in *M* between bins across all patients using repeated measures ANOVA. Second, the means across bins had to decrease from bin 1 to bin 5. Third, (uncorrected) paired t tests had to confirm that no higher number bins were significantly greater than lower number bins. Models were developed with the anterior hippocampus ipsilateral and contralateral to the seizure focus, separately. Next, to investigate temporal progression, in any findings in which network change from control was related to distance along a topology, statistics were repeated after patients were grouped into three categories based on duration of disease (described in results).

### Whole-Brain Network Change in Patients

**Figure 2.**
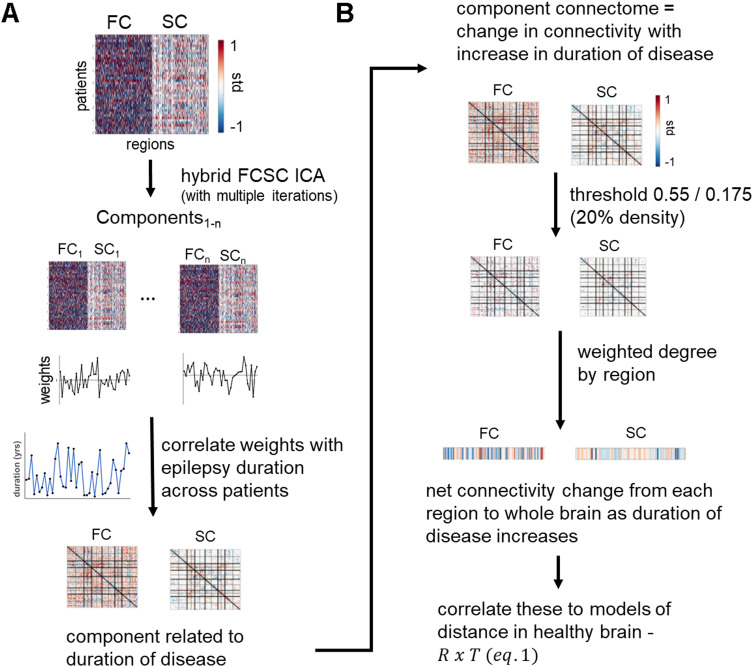
Whole-brain network change in patients. (A) A hybrid FC SC independent component analysis (ICA) was performed using age corrected functional (FC_corr_) and structural connectomes (SC_corr_) across all patients. Resulting connectome component weights were each correlated with duration of disease across patients to identify the ICA components related to duration of disease. (B) The FC and SC component connectomes related to duration of disease were thresholded, and a weighted degree to each region was computed to create vectors of region to whole-brain connectivity related to duration of disease. These vectors were then compared to the models of distance to the anterior hippocampus or along a functional gradient in the healthy brain; ant hip = anterior hippocampus; FC = functional connectivity; SC = structural connectivity.

Next, we developed a method to compare whole-brain network progression in patients as a function of distance in the healthy brain. To do this, a hybrid functional and structural connectome independent component analysis (ICA) method proposed by Amico and Goñi (Amico & Goñi, 2018) with fastICA (Hyvarinen, 1999) was implemented (Figure 2A). Joint functional-structural connectomes from each patient were created by concatenating the FC_corr_ and SC_corr_ connectomes (in units of SD from healthy age-matched controls) after transforming from left and right hemispheres to ipsilateral and contralateral to group all patients. Principal component analysis was used to reduce dimensionality of the data through decomposition and reconstruction of 90% of variance. Then, due to the nondeterministic behavior of the fastICA algorithm, it was run 40 times. The output connectome components for each run were compared, and any two component connectomes with a Pearson correlation coefficient greater than 0.8 were combined through averaging. Their associated patient weights were also averaged. This resulted in a set of combined component connectomes and patient weight vectors across all runs (Amico & Goñi, 2018). The weights for the final components were compared to duration of disease across patients to identify any FCSC components that exhibit a monotonic relationship (Spearman correlation) to increasing duration of disease. In such components, the values in the connectome reflect connections that increase (or decrease) in connectivity with increase in duration of disease. Next, to compare the connectome components to the model vectors of distance (Equation 1), they were converted to vectors representing full brain connectivity to each region in two steps (Figure 2B). First, the FC and SC connectome components were each thresholded at +/−0.55 and +/− 0.175, respectively, to remove the effect of the edges with lowest relationship to duration of disease and create a matrix of approximately 20% density. Second, a weighted degree (Rubinov & Sporns, 2010) was computed for each region to create a single value of net connectivity from each region to the whole brain. This vector of connectivity from each region to whole brain was then correlated to the models of distance in the healthy brain. Note that the ICA component regions are identified in relation to ipsilateral and contralateral to the seizure focus, whereas the model distances were computed from controls as left and right regions. Therefore, the distance to each region used in the model was the average of the distances in the left and right hemispheres. Significant correlation would suggest that a region’s connectivity change across the brain with increasing duration of disease is associated with its distance from the anterior hippocampus or across a functional gradient in the healthy brain.

## RESULTS

### Hippocampal Network Change in Patients Related to Healthy Brain Topology

**Figure 3.**
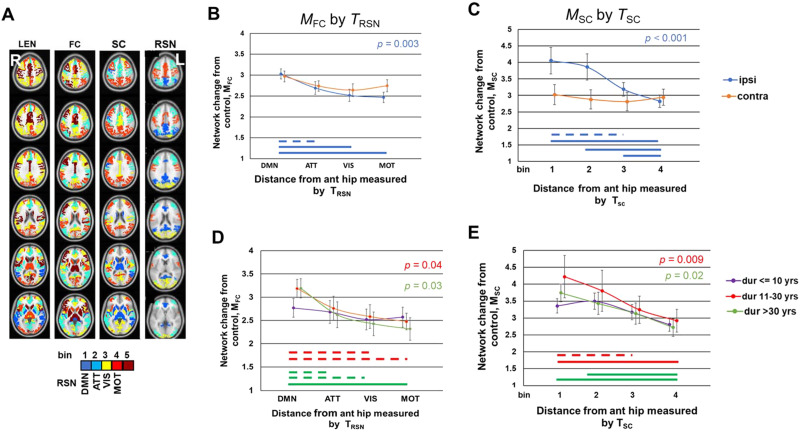
Hippocampal network change in patients related to healthy brain topology. (A) The four topologies were used to create 4 or 5 bins of regions based on their distance to the anterior hippocampus (ant hip) in the same hemisphere or along a functional network gradient. Bin 1 has the lowest streamline length (LEN), highest absolute value of functional connectivity (FC), or highest structural connectivity (SC) to the anterior hippocampus in the same hemisphere. The resting-state networks (RSN) were ranked based on a gradient of transmodal to unimodal function. (B) Ipsilateral hippocampal functional network change across all patients, *M*_*FC*_, decreases as a function of the gradient of transmodal to unimodal resting-state network, *T*_*RSN*_. (C) Ipsilateral hippocampal structural network change across all patients, *M*_*SC*_, decreases with increasing distance based on structural connectivity to the anterior hippocampus, *T*_*SC*_. (D) Hippocampal functional network change, *M*_*FC*_, as a function of the gradient of transmodal to unimodal functional resting-state network, *T*_*RSN*_, in patients grouped by duration of disease. (E) Hippocampal structural network change in patients, *M*_*SC*_, with increasing distance from the anterior hippocampus based on structural connectivity, *T*_*SC*_, in patients grouped by duration of disease. The mixed ANOVAs for parts D and E did not reveal statistical difference between the duration groups. Individual group comparisons are shown for illustration. In B–E, the p value listed refers to repeated measures ANOVA between bins. Dashed lines represent uncorrected paired t-test difference between bins with *p* = 0.05. Solid lines represent uncorrected paired *t*-test difference between bins with *p* < 0.01. The color of the *p* value listed and the lines of significance reflect the data to which they refer; ipsi = anterior hippocampus ipsilateral to seizure focus; contra = anterior hippocampus contralateral to seizure focus; DMN = default mode network; ATT = attention network; VIS = primary visual network; MOT = motor/sensorimotor network.

The Mahalanobis distance (M) was used to quantify hippocampal network change in the patients compared to controls in bins that were ranked by distance to the focus or by functional network gradient based on topology in the healthy brain (Figure 3A). Considering *M*_*FC*_, where FC denotes functional connectivity hippocampal network change in patients, four healthy brain topologies were investigated (*T*_*LEN*_, *T*_*SC*_, *T*_*FC*_, *T*_*RSN*_). Therefore, the threshold for statistical significance of the repeated measures ANOVA was 0.0125. Post hoc t tests between bins of significant models are not corrected for multiple comparisons to illustrate all relationships. For the ipsilateral anterior hippocampus, the repeated measures ANOVA using *T*_*LEN*_, *F*(4, 156) = 2.62, *p* > 0.0125, and *T*_*SC*_, F(3, 117) = 1.53, *p* > 0.0125, were not significant. Using *T*_*FC*_, there were differences between bins, repeated measures ANOVA, *F*(4, 156) = 3.63, *p* = 0.011, but the means did not decrease across bins. Using *T*_*RSN*_, there were differences between bins, repeated measures ANOVA, F(3, 117) = 5.12, *p* = 0.003, with means decreasing across bins: default mode network > attention network > primary visual network > motor/sensorimotor network. The paired t test showed that *MFC* was higher in the default mode network than the attention (*p* = 0.05), the primary visual (*p* = 0.002), and motor/sensorimotor network (*p* = 0.001) (Figure 3B). These results suggest that functional connectivity changes from the seizure focus in mTLE occur as a function of the gradient of transmodal to unimodal functional resting-state network in the healthy brain. For the contralateral anterior hippocampus, there were no significant differences between bins for any topology (repeated measures ANOVA, *p* > 0.0125).

Considering *M*_*SC*_, where SC denotes structural connectivity hippocampal network change, three healthy brain topologies were investigated (*T*_*LEN*_, *T*_*SC*_, *T*_*FC*_). The *T*_*RSN*_ bins included cross-hemispheric edges for which structural connectivity was not reliably detected. Therefore, the threshold for significance of the repeated measures ANOVA was 0.0167. As above, posthoc *t* tests between bins of significant models are not corrected for multiple comparisons to illustrate all relationships. For the ipsilateral anterior hippocampus, using *T*_*LEN*_ there was no decreasing trend across bins, only that the bin 1 (shortest streamline length) had greater *M*_*SC*_ than the others, repeated measures ANOVA, *F*(6, 152) = 6.33, *p* < 0.001; paired *t* test, bin 2: *p* = 0.02, bin 3: *p* = 0.003, bin 4: *p* = 0.008, bin 5: *p* = 0.004. Similarly, using *T*_*FC*_ the same pattern of greater *M*_*SC*_ in bin 1 (highest absolute functional connectivity) than the other bins, repeated measures ANOVA, *F*(4, 152) = 4.11, *p* = 0.003; paired *t* test, bin 2: *p* = 0.013, bin 3: *p* = 0.004, bin 4: *p* = 0.012, bin 5: *p* = 0.039. Using *T*_*SC*_, there were differences in *M*_*SC*_ between bins, repeated measures ANOVA, F(3, 114) = 9.81, *p* < 0.001; and the means decreased across the bins, with significant differences to support the trend, paired t test, bin 1 > bin 3: *p* = 0.016, bin 1 > bin 4: *p* < 0.001, bin 2 > bin 4: *p* = 0.001, bin 3 > bin 4: *p* = 0.003 (Figure 3C). These results suggest that structural connectivity changes from the seizure focus in mTLE occur as a function of structural connectivity distance to the anterior hippocampus in the healthy brain. For the contralateral anterior hippocampus, there were no significant differences between bins (repeated measures ANOVA, *p* > 0.0167).

To investigate the temporal changes, the patients were divided into three groups based on duration of disease (≤ 10 years, ‘short,’ [*n* = 15]; 11–30 years, ‘medium,’ [*n* = 14]; >30 years, ‘long,’ [*n* = 11]), and the statistics for the best model for *M*_*FC*_ and *M*_*SC*_ were recomputed using a mixed ANOVA by adding the group identification as a between-subject factor. For *M*_*FC*_ using the resting-state network topology, *T*_*RSN*_, and for *M*_*SC*_ using the structural connectivity network topology, *T*_*SC*_, the mixed ANOVAs did not detect a significant effect of bin by group interaction, *M*_*FC*_: *F*(3, 6) = 0.33, *p* > 0.05; *M*_*SC*_: *F*(3, 6) = 0.65, *p* > 0.05. However, to begin to investigate potential relationships of interest across the duration groups, individual repeated measures ANOVA were performed are reported.

For *M*_*FC*_ the resting-state network topology, *T*_*RSN*_, was used. For the short-duration group, there was no difference between the bins, repeated measures ANOVA, *F*(3, 42) = 0.28, *p* > 0.05. For the medium-duration group, the means were different between groups, repeated measures ANOVA, *F*(3, 39) = 3.17, *p* = 0.047, with the default mode network > primary visual (paired *t* test, *p* = 4 0.014) and motor/sensorimotor network (paired *t* test, *p* = 0.011). For the long-duration group, there was difference between bins, repeated measures ANOVA, *F*(3, 30) = 3.9, *p* = 0.033, with the mean of the default mode network > the attention (paired *t* test, *p* = 0.012), primary visual (paired *t* test, *p* = 0.027) and motor/sensorimotor network (paired *t* test, *p* = 0.008) (Figure 3D). These findings imply that the progression of functional connectivity alterations in patients tends to occur over time with the highest changes in regions of transmodal resting-state networks occurring in the medium- to long-durations groups.

For *M*_*SC*_ the structural connectivity topology, *T*_*SC*_, was used. For the short-duration group, there was no difference between the bins, repeated measures ANOVA, *F*(3, 39) = 1.99, *p* > 0.05. For the medium-duration group, there was difference between bins, repeated measures ANOVA, *F*(3, 39) = 4.44, *p* = 0.009, with *M*_*SC*_ in bin 1 higher than bin 3 and bin 4 (paired *t* test, *p* = 0.048 and *p* = 0.006, respectively). For the long-duration group, again there was difference between bins, repeated measures ANOVA, *F*(3, 30) = 4.63, *p* = 0.022, with *M*_*SC*_ decreasing across bins. In this case, bin 4 was less than the first and second bin (paired *t* test, bin 1: *p* = 0.008 and *p* = 0.001, respectively) (Figure 3E). Taken together, these results suggest that changes in regions most structurally connected to the presumed focus in the healthy brain tend to occur in the second and third decade of disease.

### Whole-Brain Network Change in Patients As a Function of Distance in the Healthy Brain

**Figure 4.**
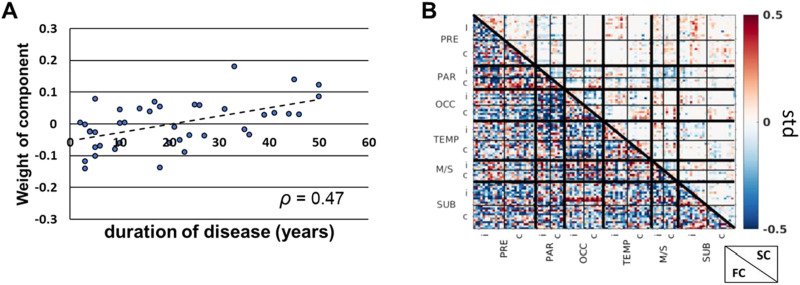
Hybrid independent component analysis (ICA) component related to duration of disease. (A) One ICA component set of weights was correlated with duration of disease across all patients (Spearman *ρ* = 0.47, *p* < 0.05). (B) The FC and SC connectome of the component that was positively correlated with duration of disease in A. Positive (negative) values indicate increased (decreased) connectivity with increased duration of disease. FC = functional connectivity; SC = structural connectivity; PRE = prefrontal; PAR = parietal; OCC = occipital; TEMP = temporal; M/S = somatosensory/motor; SUB = subcortical regions; i = ipsilateral to seizure focus; c = contralateral to seizure focus.

**Figure 5.**
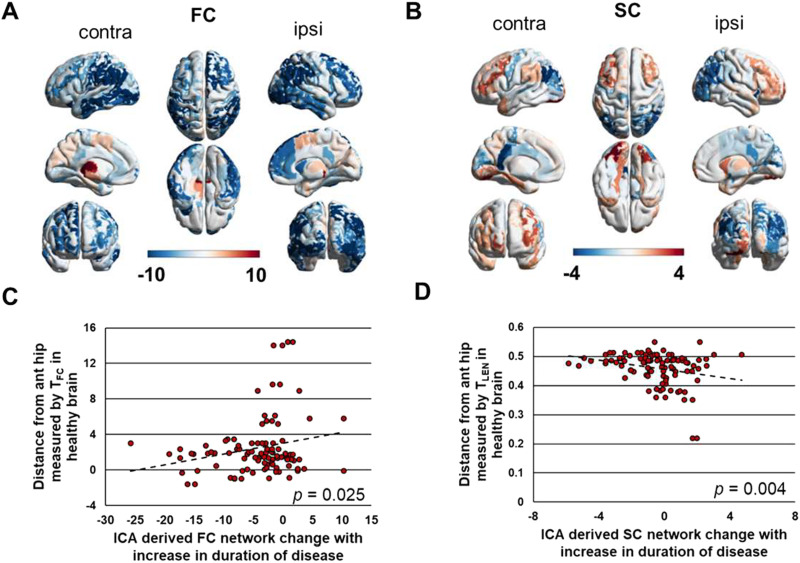
Whole-brain connectivity changes with disease duration as a function of distance in healthy brain. (A) Vector of ICA-derived regional average functional connectivity change with increasing duration of disease illustrated across the brain. (B) Vector of ICA-derived regional average structural connectivity change with increasing duration of disease illustrated across the brain. (C) ICA-derived functional connectivity change with increasing duration of disease shows a trend toward positive correlation with distance from the anterior hippocampus in the healthy brain measured by functional connectivity, *T*_*FC*_ (Pearson *r* = 0.22, *p* = 0.02). (D) ICA-derived structural connectivity change with increasing duration of disease is negatively correlated with distance from the anterior hippocampus in the healthy brain measured by streamline length, *T*_*LEN*_ (Pearson *r* = −0.26, *p* = 0.009). The colors of regions in A and B are represented by the *x*-axis in C and D, respectively. FC = functional connectivity; SC = structural connectivity; ipsi = ipsilateral to seizure focus; contra = contralateral to seizure focus; ant hip = anterior hippocampus; ICA = independent component analysis.

One component of the hybrid ICA analysis had weights that were positively correlated with duration of disease (Spearman *ρ* = 0.47, *p* < 0.05) (Figure 4A and B). This component was identified in 50% of the ICA iterations. The component FC and SC connectomes were then transformed to a vector capturing region to whole-brain connectivity (Figure 5A and B) and correlated with the four models of distance in the healthy brain. Therefore, the threshold for significance for the correlation was 0.0125.

The FC component connectome related to duration of disease correlated with the model using *T*_*FC*_ (Pearson *r* = 0.22, *p* = 0.02) (Figure 5C). This is not statically significant when correcting for multiple comparisons, but is shown for illustration as a promising relationship. When using models created with *T*_*LEN*_, *T*_*SC*_, and *T*_*RSN*_ there was no significant correlation with the connectome component (*p* > 0.05). This suggests that there is a trend that a region’s whole-brain functional connectivity change with increasing duration of disease in mTLE is a function of the region’s functional connectivity distance from the anterior hippocampus in the healthy brain. The SC component connectome related to duration of disease correlated with the model using *T*_*LEN*_ (Pearson *r* = −0.26, *p* = 0.009) (Figure 5D). No other models correlated with this connectome component (*p* > 0.05). These results imply that a region’s whole-brain structural connectivity change with duration of disease is inversely proportional to the region’s mean streamline length to the anterior hippocampus in the healthy brain.

## DISCUSSION

Focal epilepsy manifests widespread functional and structural alterations across the brain that have been well characterized (Tavakol et al., 2019). What remains unclear, however, are why are some regions more affected than others, and what is the timeline of this progression? In this work we showed that both networks connected to the seizure focus (anterior hippocampus) and global, whole-brain networks were altered in mTLE along a predictable framework defined by a given topology in the healthy brain. In addition, these changes evolved over several decades of disease duration.

The hippocampal functional network alterations in mTLE occurred across an organization of the brain related to inclusion in functional resting-state network ranked by transmodal to unimodal gradients (Margulies et al., 2016). This suggests that functional reorganization occurs with the most pronounced changes in the regions within the default mode network and those serving transmodal functions supporting higher cognitive functions, with decreasing effects into unimodal networks supporting perceptual functions. One explanation for this is that the seizure focus in the anterior hippocampus is highly connected to and sometimes considered a node in the default mode network (Buckner et al., 2008), thus reflecting more within functional network changes. Alternatively, hippocampal structural connectivity alterations in mTLE occurred as a function of distance to the anterior hippocampus in the healthy brain measured by structural connectivity. This implies that the greatest structural network changes occur in patients in regions more structurally connected to the region of the seizure focus and decrease as structural connectivity to the focus decreases in healthy controls.

When investigating the temporal characteristics of these changes in hippocampal networks, we found no difference between groups based on duration of disease. Closer examination of patterns of change in each group, however, revealed that the trends in both functional and structural networks suggest that the patterns related to the focus may develop later in the disease. This is interesting because it is counter to our presumption that changes occur close to the focus first and then spread outward later. But, this question will most likely require larger datasets to confirm.

The global whole-brain network to each region was investigated through the identification of a joint whole-brain functional and structural connection pattern related to duration of disease that was then compared to models of healthy brain architecture. Results showed a preliminary trend that functional network changes related to duration of disease increased as functional connectivity to the anterior hippocampus in the healthy brain increased. Structural network changes related to duration of disease decreased as a function of increasing distance from the anterior hippocampus measured by streamline length in the healthy brain. This means that regions with the greatest changes over the duration of disease had the highest functional connections or shortest streamline length to the presumed seizure focus in the anterior hippocampus in the same hemisphere.

Overall, it is not completely surprising that hippocampal functional network changes are related to a functional architecture, while structural network changes are related to a structural network architecture in the healthy brain. The potential consequences of this difference in architectural framework are interesting. First, these results may imply that interventions that target the seizure focus will have structural effects on those regions directly connected to the focus by white matter tracts, while functional effects may be more widespread across the brain in regions not physically connected to the focus. Second, structural (physical) changes in white matter may take longer to occur than functional changes, and so functional network changes may be detected prior to structural network connectivity. Although we did not detect this difference in this study, the magnitude of the functional network changes were approximately three times those of the structural changes when measured in standard deviations from age-matched controls, which is consistent with this idea.

There are potential clinical implications of these findings. Network information has emerged as a potential predictor of treatment outcome (Morgan et al., 2017; Gleichgerrcht et al., 2018; Taylor et al., 2018), and understanding how and when networks evolve could identify windows of time when specific treatments may be most effective. In a similar way, it may be possible to predict widespread longitudinal postsurgical network changes to the resected area to predict long-term postsurgical outcomes (Morgan et al., 2019). In addition, cognitive and behavioral impairments in mTLE and their changes over time (Helmstaedter & Kockelmann, 2006) may be better understood by their relation to network reorganization (Park et al., 2017). Finally, this work may provide a framework for investigations to predict progression in other focal epilepsies.

This study could be improved in several ways. First, a longitudinal study would provide better temporal data than this cross-sectional study. Second, these analyses did not control for age of onset of mTLE. In our cohort, age of onset was significantly negatively correlated with duration of disease (Pearson *r* = −0.59, *p* < 0.001), but the medium- and long-duration groups were not different, ANOVA *F*(2, 37) = 8.2, *p* = 0.001; *t*-test medium vs. long duration *p* > 0.05; all other *t* tests *p* < 0.05. Thus, duration is similar to, but not an exact proxy, for age of onset changes. Third, we also did not control for variations in seizure type or frequency. This analysis would require a much a larger cohort in which the complete analyses could be performed on sets of patients with more homogeneous seizure frequencies within the set. Similarly, we did not control for variations in medication use. While all patients were on medication, to address this thoroughly would require analyses of subsets of patients on the same or similar medications. In addition, a larger, more balanced cohort of right and left mTLE patients would allow for separate investigations rather than the ipsilateral/contralateral pooling performed here. These are important potential confounds that would need a larger or more homogeneous sample size to address. Finally, the investigations were based on associations, while causation and other effects for these changes require further studies.

In conclusion, these findings support the idea that widespread network changes in mTLE occur along specific pathways that can be predicted by healthy brain architecture over the duration of disease. The greatest changes in hippocampal functional networks in mTLE occurred in regions of transmodal functional resting-state networks, with decreasing changes in regions involved in unimodal functional networks. Other networks studied had greatest change in regions with shortest distance to the seizure focus, with decreasing changes as the distance from the focus increased. Distance was quantified by functional connectivity, structural connectivity, or streamline length to the anterior hippocampus (presumed seizure focus) in the healthy brain. Overall, this work presents a framework of spatiotemporal network progression over duration of disease related to the seizure focus and healthy brain architecture that may be used to predict individual network evolution in focal epilepsy.

## AUTHOR CONTRIBUTIONS

Victoria L. Morgan: Conceptualization; Data curation; Formal analysis; Funding acquisition; Investigation; Methodology; Project administration; Resources; Supervision; Validation; Visualization; Writing – original draft; Writing – review & editing. Graham W. Johnson: Formal analysis; Methodology. Leon Y. Cai: Formal analysis; Methodology. Bennett A. Landman: Formal analysis; Methodology; Software. Kurt G. Schilling: Methodology. Dario J. Englot: Conceptualization; Data curation; Formal analysis; Funding acquisition; Investigation; Methodology; Writing – review & editing. Baxter P. Rogers: Formal analysis; Investigation; Methodology; Writing – review & editing. Catie Chang: Conceptualization; Formal analysis; Funding acquisition; Investigation; Methodology; Writing – review & editing.

## FUNDING INFORMATION

Victoria L. Morgan, National Institute of Neurological Disorders and Stroke (http://dx.doi.org/10.13039/100000065), Award ID: NS075270. Victoria L. Morgan, National Institute of Neurological Disorders and Stroke (http://dx.doi.org/10.13039/100000065), Award ID: NS108445. Victoria L. Morgan, National Institute of Neurological Disorders and Stroke (http://dx.doi.org/10.13039/100000065), Award ID: NS110130. Dario J. Englot, National Institute of Neurological Disorders and Stroke (http://dx.doi.org/10.13039/100000065), Award ID: NS097618. Dario J. Englot, National Institute of Neurological Disorders and Stroke (http://dx.doi.org/10.13039/100000065), Award ID: NS112252.

## TECHNICAL TERMS

Focal epilepsy: When seizures originate in one region of the brain.
Structural connectivity: Brain region interaction measured by integrity of diffusion of water across white matter using MRI.
Functional connectivity: Temporal correlations of spontaneous low-frequency blood oxygenation signal measured by MRI.
Electroencephalography (EEG): The measurement of electrical activity in your brain using noninvasive electrodes attached to your scalp, but can also use invasive probes.
Positron emission tomography (PET): Noninvasive functional imaging measure of metabolic processes in the body using radioactive tracers.
Hippocampal sclerosis: Anatomic hallmark of mesial temporal lobe epilepsy characterized by severe neuronal cell loss and gliosis in the hippocampus.
Mahalanobis distance: The measurement of distance between objects that takes into account the correlation in the data by calculating the inverse of the variance-covariance matrix of the data of interest.
Independent component analysis: A statistical method for identifying sources (components) that are linearly mixed in a measured signal.
Functional resting-state network: Regions of the brain with correlated spontaneous oxygenation fluctuations measured by functional MRI; usually denotes groups of regions forming networks for integrated task performance.
